# Advanced baseline immunosuppression is associated with elevated levels of plasma markers of fungal translocation and inflammation in long-term treated HIV-infected Tanzanians

**DOI:** 10.1186/s12981-021-00381-9

**Published:** 2021-08-26

**Authors:** Godfrey Barabona, Macdonald Mahiti, Mako Toyoda, Doreen Kamori, Salim Masoud, George P. Judicate, Bruno Sunguya, Eligius Lyamuya, Takamasa Ueno

**Affiliations:** 1 Joint Research Center for Human Retrovirus Infection, Kumamoto University, 2-2-1 Chuo-ku, Honjo, Kumamoto, 860-0811 Japan; 2 Muhimbili University of Health and Allied Sciences, Dar es Salaam, Tanzania

**Keywords:** HIV/AIDS, chronic inflammation, HIV treatment in low-income counties, 1-3-β-d-Glucan

## Abstract

**Background:**

For over a decade, antiretroviral therapy (ART) in resource-limited countries was only recommended for patients with advanced HIV disease. We investigated this group of patients in order to determine any relationship between degree of immunosuppression during treatment initiation and the subsequent levels of inflammatory biomarkers, reservoir size and plasma marker of fungal translocation after achieving long-term virological control.

**Methods:**

We analyzed 115 virally suppressed (female 83.5%) and 40 untreated (female 70%) subjects from Dar es Salaam, Tanzania. The size of HIV latent reservoir (proviral DNA copy) was determined using quantitative PCR. Inflammatory biomarkers; IL-6, IL-10, and soluble CD14 (sCD14), were measured using multiplex cytometric beads array. Antibody titers for Cytomegalovirus (CMV) and Epstein Barr virus (EBV), plasma level of 1-3-beta-d-Glucan (BDG) was measured using ELISA. High-sensitivity C-reactive protein (hsCRP) was measured using nephelometric method.

**Results:**

The median age was 36 (IQR 32-44) and 47 (IQR 43–54) years in untreated and virally suppressed patients respectively. Median duration of treatment for virally suppressed patients was 9 years (IQR 7–12) and median baseline CD4 count was 147 cells/mm^3^ (IQR 65–217). Virally suppressed patients were associated with significantly lower plasma levels of IL-10, sCD14 and BDG (P < 0.05) when compared to untreated patients. However, plasma level of IL-6 was similar between the groups. Baseline advanced level of immunosuppression (CD4 < 100cells/cm^3^) was associated with significantly higher plasma level of IL-6 (*P* = 0.02), hsCRP (*P* = 0.036) and BDG (*P* = 0.0107). This relationship was not seen in plasma levels of other tested markers. Degree of baseline immunosuppression was not associated with the subsequent proviral DNA copy. In addition, plasma levels of inflammatory marker were not associated with sex, CMV or EBV antibody titers, treatment duration or regimen.

**Conclusions:**

Our data suggest that advanced immunosuppression at ART initiation is associated with severity of inflammation and elevated fungal translocation marker despite long term virological control. Further studies are needed to evaluate the potential increased burden of non-AIDS comorbidities that are linked to elevated inflammatory and fungal translocation markers as a result of the policy of HIV treatment at CD4 count < 200 cells/cm^3^ implemented for over a decade in Tanzania.

**Supplementary Information:**

The online version contains supplementary material available at 10.1186/s12981-021-00381-9.

## Introduction

Antiretroviral therapy (ART) is responsible for the reduction in AIDS related deaths and improvement of life expectancy among people living with HIV (PLHIV). However, morbidity and mortality from conditions like metabolic syndrome, non AIDS defining cancers, cardiovascular diseases and neurocognitive disorders are disproportionally common among PLHIV [1–4]. These conditions (collectively referred as non-AIDS comorbidities) are closely linked to inflammation and immune activation. Indeed, plasma levels for markers of systemic inflammation, and monocyte activation are chronically elevated in PLHIV and have been independently associated with non-AIDS comorbidities [1–4]. The exact mechanisms involved in chronic inflammation in PLHIV remain elusive. However, current evidence suggests that dysbiosis and gut microbiome translocation in HIV infection is largely responsible for the persistent inflammation and immune dysregulation. In addition HIV replication and chronic viral co infection have also been shown to contribute to this phenomenon [5].

Generally, ART may result in decline of the plasma levels of some inflammatory markers linked to non-AIDS comorbidities. However, this has been largely demonstrated in studies of PLHIV initiating treatment in acute stages of HIV infection and/or in moderate immunosuppression (CD4 count > 200cell/cm^3^ ) [6, 7] effectively leaving a data gap on the population of PLHIV initiated treatment at advanced immunosuppression. This population is important in resource limited countries such as Tanzania, as it forms a significant proportion of PLHIV on treatment as a result of late presentation to care [8–10] and a WHO backed policy where ART was only for those with advanced immunosuppression (CD4 count < 200cell/cm^3^) [11]. On the other hand, advanced immunosuppression in PLHIV is associated with increased fungal colonization of the gut [12, 13], which in theory could increase fungal translocation into the circulation. In fact, growing evidence indicate that gut fungal translocation based on plasma level of 1-3-beta-d-glucan (BDG) is elevated in PLHIV and has a role in chronic inflammation [14–17]. Thus understanding the consequences of severe immunosuppression at treatment initiation to the subsequent inflammation status in PLHIV is critical.

In this study we recruited PLHIV on stable ART that initiated treatment at advanced immunosuppression as well as untreated patients from Tanzania. We then determined whether ongoing severity of inflammation and fungal translocation could be linked to the degree of immunosuppression at baseline despite long-term treatment.

## Materials and methods

### Study participants and ethical considerations

In this cross sectional study, we recruited a total of 155 HIV-infected adults between January and August, 2018 from two care and treatment clinics in Dar es Salaam, Tanzania. Of these, 115 were patients that were initiated on treatment at advanced HIV disease and on stable long term ART with evidence of virological suppression (VL < 40 copies/ml) within 2–3 years of recruitment for the study. The rest (n = 40) were untreated newly diagnosed HIV-infected patients. All patients were conveniently enrolled into the study and their demographics and clinical data were collected using a structured questionnaire. Patients with symptoms of acute bacterial, parasite or viral infections were excluded from the study. Prior to enrolment to the study, a written informed consent was obtained from each patient.

### Sample collection and storage

Venous blood samples were collected in EDTA coated tubes. Plasma was separated by centrifugation followed by PBMC isolation from cellular sediments using the Ficoll density gradient method. Both plasma and PBMCs samples were immediately stored in aliquots at − 80 °C within three hours of sample collection.

### Quantitation of markers for Inflammation, fungal translocation and immune activation

The inflammatory markers and soluble immune activation profile were determined using customized Cytometric Bead Array (CBA) (BD Biosciences, San Jose, California). Individual plex assay for IL-6, IL-10 and sCD14 were used to configure a multiplex system for quantitation of the targeted biomarkers simultaneously as per manufacturer instructions. Briefly, diluted plasma samples and standards (10 dilutions) were incubated with specific capture beads for 1 h at room temperature. The mixture was incubated for additional 2 h after adding the detection reagent. After a final wash, beads were acquired in a BD FACS Canto II, previously set up for the BD CBA Flex Set. For each cytokine, at least 400 beads were acquired per sample. Data analysis was performed using the FCAP Array Software (BD Biosciences). Plasma level of 1-3-beta-d-glucan (BDG) which is a cell wall component of most fungal species and highly immunogenic was quantified using enzyme-linked immunosorbent assay (ELISA) (Quickdetect Biovision CA USA). High-sensitivity C-reactive protein (hsCRP) was measured by nephelometric analyzer *N*-latex CRP II (CardioPhase hsCRP- Mississauga-Canada).

### Quantitation of Total HIV proviral DNA load

HIV proviral DNA quantitation was performed as previously described [18]. Briefly, cellular DNA was extracted from sample with greater than 5 × 10^6^ PBMCs (14 samples < 5 × 10^6^ cells because of availability) using QIAamp DNA mini kit (QIAGEN) according to the manufacturer’s specifications. Total DNA was eluted in 100µL of DNase-free water and stored at − 80 °C until use. Quantitative real-time PCR analyses was carried out with Premix Ex Taq (Takara-bio, Japan) and qPCR reactions was performed by using LightCycler 96 System (Roche Diagnostics GmbH, Mannheim, Germany). To quantify the cell number, qPCR was performed simultaneously for *ccr5* gene copy number as previously described [18, 19]. The HIV-1 proviral copy number was calculated per 10^6^ PBMCs. Primers and probes used were as follows: LTR forward, 5ʹTACTGACGCTCTCGCACC3ʹ; LTR reverse, 5ʹTCTCGACGCAGGACTCG3ʹ; LTR probe, 5ʹFAM-CTCTCTCCTTCTAGCCTC-MGB3ʹ; CCR5 forward, 5ʹATGATTCCTGGGAGAGACGC3ʹ; CCR5 reverse, 5ʹAGCCAGGACGGTCACCTT3ʹ; CCR5 probe, 5ʹFAM-AACACAGCCACCACCCAAGTGATCA-MGB3ʹ.

### Statistical analysis

Measures of central tendencies and dispersion (mean, median, range) were used to summarize data for plasma viral load, inflammatory biomarker, provial DNA, age and duration of treatment. Non parametric Mann- Whitney U test was employed to compare medians of each variable between virally suppressed patients and untreated group. Spearman rank correlation test was used to assess relationships between inflammatory markers and the associated factors. Linear regression was employed to analyze the data obtained from the standard curve of qPCR. A P-value of < 0.05 was considered significant. All data analyses were done in SPSS statistics software 21.0 and Graphpad prism ver7.

## Results

### Clinical characteristics of participants

Among the recruited patients in this study, 115 were virally suppressed with a media age of 47 years and 83.5% of which were female. In untreated patients (n = 40) median age was 36 years and 70% were female. The median duration of ART among virally suppressed patient was 9 years and started treatment with median baseline CD4 count of 147 (IQR 65–217) (Table 1). Majority (81.7%) of patients were maintained on Nonnucleoside Reverse Transcriptase Inhibitor (NNRTI)-based first-line regimen. Virally suppressed patients displayed wide range of proviral DNA load with median of 473 (IQR 236–795) copies/10^6^ PBMCs, which was significantly lower compared to that of untreated patients (*P* < 0.001). Almost all of the virally suppressed patients were positive for both Epstein Barr virus (EBV) and Cytomegalovirus (CMV) IgG antibody. We also observed that despite the similar route of transmission, hepatitis C co-infection was uncommon, present in only two (1.1%) of patients (Table 1).

**Table. 1:** Demographic and clinical characteristics of recruited subjects

	Untreated (n = 40)	Virally suppressed (n = 115)	P value
**Demographic data **			
Median age in years (IQR)	36 (32–44)	47 (43–54)	< 0.0001^a^
Gender -female n (%)	28 (70)	96 (83.5)	0.0019^b^
**Clinical data **			
Median Years on treatment (IQR)	N/A	9 (7–12)	
Missing data		n = 26	
Median current CD4 count cells/cm^3^ (IQR).	N/A	503 (329–633)	
Missing data		n = 7	
Median baseline CD4 count cells/cm^3^ (IQR)	299 (160–486)	147 (65–217)	< 0.0001^a^
Missing data	n = 5	n = 41	
**Current ART regimen (%)**			
NNRTI based first line regimen	N/A	94(81.7)	
PI based second line regimen	N/A	20 (17.4)	
Missing data		1 (0.9)	
** HIV quantification **			
Median Viral load copies/ml (IQR)	45,837 (7325-176350)	N/A	
Median proviral load copies/million PBMCs(IQR)	1422 (571–2831)	464.3 (235–758)	< 0.0001^a^
** Serology-positive (%) **			
Hepatitis C		2 (1.73)	
Epstein Barr virus		115 (100)	
Cytomegalovirus		107 (93.0)	

*NA * not applicable, *IQR *interquartile range antiretroviral therapy, *PBMCs *peripheral blood mononuclear cells

^a^ Chi square test, ^b^ Mann–Whitney U test

### Plasma BDG, and its relationship with markers of systemic inflammation

**Fig. 1:**
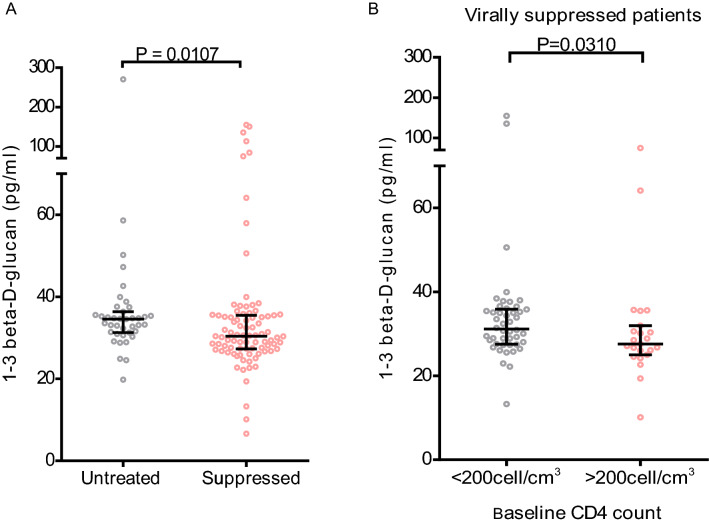
Comparison of plasma level of fungal translocation marker 1-3 beta-d-glycan (BDG) among HIV infected subjects. **A** Comparison across on ART treatment status, **B** Comparison among virally suppressed subject based on degree of immunosuppression during treatment initiation. Horizontal lines represent median and interquartile range. Each dots represent value of BDG of individual subjects. P values was obtained by Mann–Whitney U test

All tested samples had detectable plasma BDG with the median (range) of 31pg/ml (6.64–270.65). When compared between virally suppressed and untreated patients, plasma BDG levels was slightly but statistically significantly higher in untreated patients (P = 0.0107) (Fig. 1a). To assess the effect of baseline immunosuppression on ongoing plasma levels of BDG, we compared BDG levels in patients whose baseline CD4 count were above vs. below 200cells/cm^3^. Among virally suppressed patients BDG was significantly higher in patients with baseline CD4 count < 200cells/cm^3^ (P = 0.0310) (Fig. 1b). In contrast, there were no significant differences in plasma BDG when similar analysis was performed based on recent CD4 count in virally suppressed patients or among untreated patients. We found no significant relationship between plasma BDG and markers of inflammation (IL-6, IL-10 and hsCRP) or monocyte activation (sCD14) (Table 2).

**Table 2:** Rank correlation between plasma levels of inflammatory markers

	IL-6	IL-10	sCD14	BDG	hsCRP
Untreated patients					
IL-6	1	**0.38** **(0.015)**	− 0.17 (0.3088)	0.038 (0.818)	
IL-10		1	− 0.19 (0.2523)	− 0.013 (0.936)	
sCD14			1	− 0.294 (0.069)	
Virally suppressed					
IL-6	1	− 0.05 (0.646)	**0.30** **(0.007)**	− 0.042 (0.709)	**0.38** **(0.0001)**
IL-10		1	**− 0.37** **(0.001)**	0.20 (0.074)	− 0.02 (0.868)
sCD14			1	− 0.151 (0.223)	0.02 (0.836)
BDG				1	0.086 (0.430)
hsCRP					1

Spearman rank correlation r (P value)

*BDG* 1-3 beta-d-glucan, *hsCRP* highly-sensitive C-reactive protein, *sCD14* soluble cluster of differentiation 14, Bold values indicate statistically significant correlations

### Inflammatory markers in virally suppressed and untreated patients

**Fig. 2:**
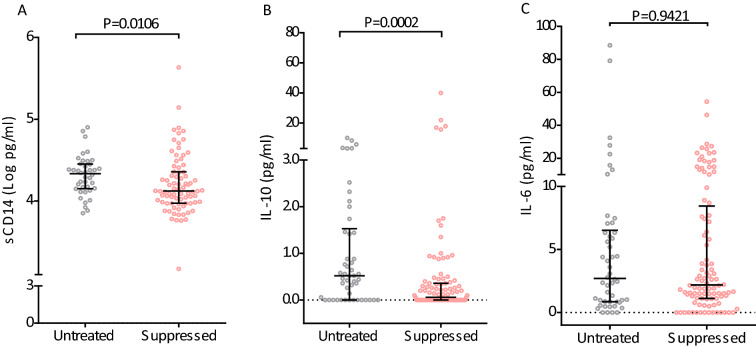
Comparison of plasma levels of markers of inflammation and immune activation in HIV infected subjects based on ART treatment status. **A** Plasma levels of monocyte activation marker–sCD14, **B** Interleukin-10, and **C** Interleukin-6. Horizontal lines represent median and interquartile range. Each dots represent value of marker of individual subjects. For comparison purposes IL-10 and IL-6 values below detection limit were regarded as zero (0). P values was obtained by Mann–Whitney U test

Among the three (IL-6, IL-10 and sCD14) inflammatory markers that we tested and compared between untreated and virally suppressed patients, IL-10 and sCD14 were significantly elevated among untreated patients (P < 0.02) (Fig. 2a, b). However, there was no statistical difference in the plasma levels of IL-6 between the two groups despite long-term ART in virally suppressed patients (P > 0.9) (Fig. 2c).

### Relationship between degree of immunosuppression and level of inflammatory markers

**Fig. 3:**
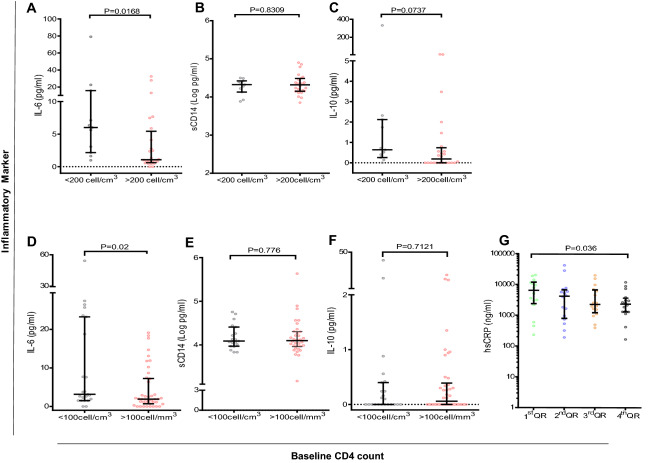
Effect of degree of immunosuppression at baseline to the ongoing inflammation and immune activation among untreated and virally suppressed HIV subjects. Data depicts comparison of plasma levels of inflammatory or immune activation marker between different classifications of degree of immunosuppression at baseline (during treatment initiation). **A** Interleukin-6, **B** monocyte activation marker-sCD14, **C** interleukin-10 in untreated subjects with baseline CD4 count below vs. above 200cells/cm^3^ in virally suppressed subjects’ comparison of markers, **D** interleukin-6, **E** monocyte activation marker-sCD14, and **F** Interleukin-10; in subjects with CD4 counts below vs. above 100cells/cm^3^. **G** Comparison of plasma level of high sensitive C- reactive protein (hsCRP) based of quartile of baseline CD4 count, only P value < 0.05 is shown. For comparison purposes IL-10 and IL-6 values below detection limit were regarded as zero (0). All P values was obtained by Mann–Whitney U test

We next analyzed the relationship between baseline CD4 count and the ongoing levels of inflammation. We found that there was no statistically significant correlation between baseline CD4 count and level of IL-6, sCD14 or hsCRP in both untreated and virally suppressed patients whose baseline data were available (Table 3). However, untreated patients with baseline CD4 count < 200 cells/cm^3^ had significantly elevated levels of IL-6 compared to those with higher baseline CD4 count (P = 0.0168) (Fig. 3a).

**Table. 3:** Rank correlation between inflammatory markers and patients characteristics

	IL-6	IL-10	sCD14	BDG	hsCRP
Untreated					
Age	**0.40 (0.014)**	0.10 (0.559)	0.15 (0.368)	− 0.006 (0.973)	
Baseline viral load	**0.56 (0.001)**	**0.64 (0.0001)**	− 0.26 (0.157)	− 0.041 (0.831)	
BMI	0.20 (0.246)	− 0.15 (0.560)	0.20 (0.261)	− 0.045 (0.801)	
Baseline CD4 count	− 0.33 (0.050)	− 0.34 (0.765)	0.17 (0.315)	0.009 (0.958)	
Virally suppressed					
Age	− 0.02 (0.811)	0.04 (0.676)	− 0.08 (0.504)	− 0.149 (0.169)	**0.19 (0.041)**
Duration of treatment	0.05 (0.699)	− 0.06 (0.609)	0.23 (0.076)	− 0.094 (0.384)	0.00 (0.992)
Baseline CD4 count	− 0.23 (0.063)	0.06 (0.618)	− 0.05 (0.736)	− 0.175 (0.151)	− 0.21 (0.068)
Recent CD4 count	− 0.02 (0.884)	0.00 (0.978)	0.04 (0.728)	− 0.062 (0.569)	0.14 (0.148)
Proviral DNA load	− 0.01 (0.932)	− 0.10 (0.335)	0.05 (0.697)	− 0.087 (0.425)	0.10 (0.305)
EBV IgG titers	− 0.06 (0.588)	0.02 (0.806)	0.12 (0.286)	− 0.083 (0.448)	0.08 (0.409)
CMV IgG titers	0.02 (0.815)	− 0.11 (0.292)	0.12 (0.296)	− 0.024 (0.827)	0.03 (0.752)

Spearman correlation coefficient r (P value)

*BDG*1-3 beta-d-glucan, *BMI* body mass index, *CMV* Cytomegalovirus, *EBV* Epstein Barr virus, *hsCRP* highly-sensitive C-reactive protein, *sCD14* soluble cluster of differentiation 14,  Bold values indicate Correlations with P value <0.05

In the similar analysis among virally suppressed patients, there were no differences between groups (Additional file 1: Figure S1). However, it was found that patients with baseline CD4 count < 100 cells/cm^3^ exhibited significantly elevated levels of IL-6 compared to those with higher baseline CD4 count (P = 0.02) (Fig. 3d). We observed a similar trend in the level of hsCRP where, virally suppressed patients in the lowest quantile of baseline CD4 count had higher levels compared to those in the highest quantile (*P* = 0.036) (Fig. 3g). This relationship was not seen in plasma levels of sCD14 or IL-10 in both groups (Fig. 3e and f). Of note, there were no significant differences in patient characteristics (age, sex, duration of treatment, treatment regimen, current CD4, proviral DNA load) between the discussed CD4 categories (Additional file 2: Table S1).

### Rank correlation among markers of inflammation, HIV infection, viral co-infections and clinical characteristics

Between plasma levels of IL-6, IL-10 and sCD14 among untreated patients, a significant positive correlation was observed only between IL-6 and IL-10 (P = 0.015). In contrast, among virally suppressed patients, plasma levels for sCD14 were significantly correlated with IL-6 and IL-10 (P < 0.01) (Table 2), indicating a relationship between monocyte activation and inflammation in virally suppressed patients but not in untreated patients.

Plasma viral load in untreated patients was significantly correlated with IL-6 and IL-10 (P < 0.001), but not sCD14 (p > 0.15) (Table 3), suggesting that the active viral replication, but not monocyte activation, is related with inflammation in untreated patients. However, presence of detectable plasma viral load was not associated with higher levels of inflammatory markers in virally suppressed patients (P > 0.05). In addition, proviral DNA load in virally suppressed patients was not associated to any of the tested inflammatory markers. Although viral co-infection has been associated with chronic inflammation and size of latent reservoir in HIV infection [5, 20, 21] we found no statistically significant relationship between antibody titers for CMV or EBV and inflammatory markers or proviral DNA load among virally suppressed patients (Table 3). We found no significant correlation between proviral DNA load and the baseline degree of immunosuppression during ART initiation or the duration of ART treatment (Table 3).

## Discussion

In the past two decades, intentional efforts have been made to expand access to ART in resource limited countries including Tanzania. As a result, individuals on long-term suppressive ART are increasingly becoming the dominant population among PLHIV [22]. However, a substantial proportion of this population was initiated on ART at an advanced level of immunosuppression due to earlier standard of care recommendations and late presentation to care. In this study we analyzed a subset of this population of PLHIV and found that despite long term ART, advanced immunosuppression at treatment initiation was associated with elevated levels of markers of fungal translocation and systemic inflammation.

Contribution of fungal translocation in chronic inflammation and development of non-AIDS comorbidity among PLHIV is increasingly being supported by recent reports. Multiple studies have reported association between plasma level of BDG and that of inflammatory cytokines, monocyte activation markers as well as co-morbidities like cardiovascular diseases and neurocognitive impairment in PLHIV [14–17]. Although fungal species make a small percentage of overall gut microbiota, the disruption of gut microbiota and depletion of CD4^+^ T cells in advanced HIV infection [12] may pave the way for fungal overgrowth and exacerbate the role of fungal translocation in the pathogenesis of chronic inflammation. The effect of ART on plasma levels of BDG remains inconclusive; one longitudinal study showed over 1.5-fold increase in BDG after 24 or more months of treatment [23] while another study found a stable level of BDG after 24 months of treatment [24]. In our study BDG was significantly higher in plasma of PLHIV who were initiated treatment at very low CD4 count (< 200cells/cm^3^) despite being on treatment for a median of 9 years. In contrast, there was no difference in BDG levels when groups were compared based on recent CD4 count, indicating that degree of immunosuppression during ART was not associated with BDG level in our study patients. Therefore, our finding together with those of others suggest persistent fungal translocation despite suppressive ART, the severity of which depends on the degree of immunosuppression at ART initiation. These findings are consistent with the hypothesis that advanced HIV disease favors further fungal colonization of the gut and higher degree of gut epithelial destruction which in turn favor systemic translocation whose implications are long term even after treatment. Further studies exploring more markers of microbial translocation and gut integrity are therefore needed to investigate this mechanism.

In this study we did not find significant association between BDG and IL-6 or sCD14 which was demonstrated in some studies [15, 23, 24]. However, our results are similar to other studies which did not find relationship between these markers [14, 17]. This may be due to the differences in the population of PLHIV across the studies. Of note, our study did not investigate all inflammatory markers that have been associated with plasma level of BDG. Therefore, further studies on role of BDG and chronic inflammation across different PLHIV populations are warranted.

IL-6 and hsCRP are sensitive markers of systemic inflammation. Several studies have linked elevation of these markers with non-AIDS comorbidities including metabolic syndrome, neurodegenerative disorders and cardiovascular diseases in PLHIV [5]. Our findings show similar IL-6 levels between untreated and virally suppressed patients. This may suggest that other factors other than active viral replication are responsible for the elevated levels of IL-6. Importantly, it also appears that severe immunosuppression at treatment initiation and not the degree of immunosuppression during treatment is associated with elevated plasma levels of both IL-6 and hsCRP despite long-term treatment. These findings highlight the potential risk of non-AIDS comorbidities due to heightened inflammation among patients initiating treatment after developing severe immunosuppression.

There are several approaches proposed for managements of chronic inflammation and immune activation in PLHIV. However, results from clinical trials on the use of conventional and unconventional anti-inflammatory agents as well as probiotics [25, 26], are promising but remain unsatisfactory [27–30]. In this study, we observed that relationship between markers of inflammation varied with treatment status. For example, a marker of monocyte activation (sCD14) was only correlated with IL-6 and IL-10 in virally suppressed patients and not in untreated patients. This may suggest (at least in our study participants) that factors and mechanisms for maintaining chronic inflammation in PLHIV may not entirely be the same before and after long-term treatment. Understanding of the underlying mechanisms will be crucial for the fine tuning of management of inflammation in PLHIV in different phases of HIV infection.

Protein transcripts from both intact and defective proviruses have been shown to contribute to systemic inflammation [31]. On the other hand residual inflammation may contribute to maintenance and expansion of the latent reservoir [32]. In this study we did not find significant association between latent reservoir size (estimated by proviral DNA copy) and the tested markers of inflammation or immune activation. These findings are similar to those described in other studies [33, 34]. Thus, from our data it does not appear that HIV proviral DNA copy is either influencing or being influenced by inflammation. It is noteworthy that we did not find relationship between inflammation markers and sex, IgG titers for EBV and CMV, treatment regimen or duration of treatment.

Our study has some limitations including a small sample size and lack of HIV negative control group. In addition, our analysis were not adjusted for multiple comparison. Although we clinically excluded active acute infection before recruiting our study participants, this method does not guarantee exclusion of ongoing subclinical acute infections, which may interfere with the overall inflammation profile of study participants. The cross-sectional study approach did not allow us to test the temporal and causal relationship between the markers of inflammation, immune activation and fungal translocation. In addition, our study is not able to confirm the source of BDG as fungal colonization in other organs other than the gut may contribute to levels of BDG in the plasma. Nevertheless, the strength of our study include a broad analysis of factors associated with chronic inflammation in PLHIV including chronic viral co infection, latent reservoir size and markers of fungal translocation, inflammation and immune activation in patients stating ART at extremely low CD4 counts whose data are barely available.

In conclusion our data suggest that initiation of ART at advanced immunosuppression state is associated with severity of inflammation and elevated fungal translocation marker among PLHIV. This could imply that the policy of HIV treatment at severe immunosuppression implemented for over a decade by lower income countries may have consequences on the burden of non-AIDS comorbidities that have been linked to elevated inflammatory and fungal translocation markers. Therefore, further studies on the implication of elevated fungal translocation markers and inflammation on morbidity in PLHIV initiated treatment at an advanced level of immunosuppression in Tanzania and other lower income settings are needed. In addition, addressing the gaps in the case finding and execution of the current test and treat policy [35] remain important in ensuring that PLHIV actually are identified and treated at early stages of HIV disease.

## Supplementary Information

**Additional file 1: Figure S1.** Effect of degree of baseline immunosuppression to plasma level of inflammatory and immune activation markers among virally suppressed HIV patients.

**Additional file 2: Table S1.** Patients characteristics according to baseline CD4 counts

## Data Availability

The corresponding author can provide additional information/data set used and/or analysed during the current study upon reasonable request.
