# Supplementary material for: Advanced baseline immunosuppression is associated with elevated levels of plasma markers of fungal translocation and inflammation in long-term treated HIV-infected Tanzanians
Source: AIDS Res Ther. 2021 Aug 26;18:55. doi: 10.1186/s12981-021-00381-9 (PMC8394626; doi:10.1186/s12981-021-00381-9)
Supplement: Supplementary file 1 — Additional file 1: Figure S1. Effect of degree of baseline immunosuppression to plasma level of inflammatory and immune activation markers among virally suppressed HIV patients. [file 12981_2021_381_MOESM1_ESM.pdf]

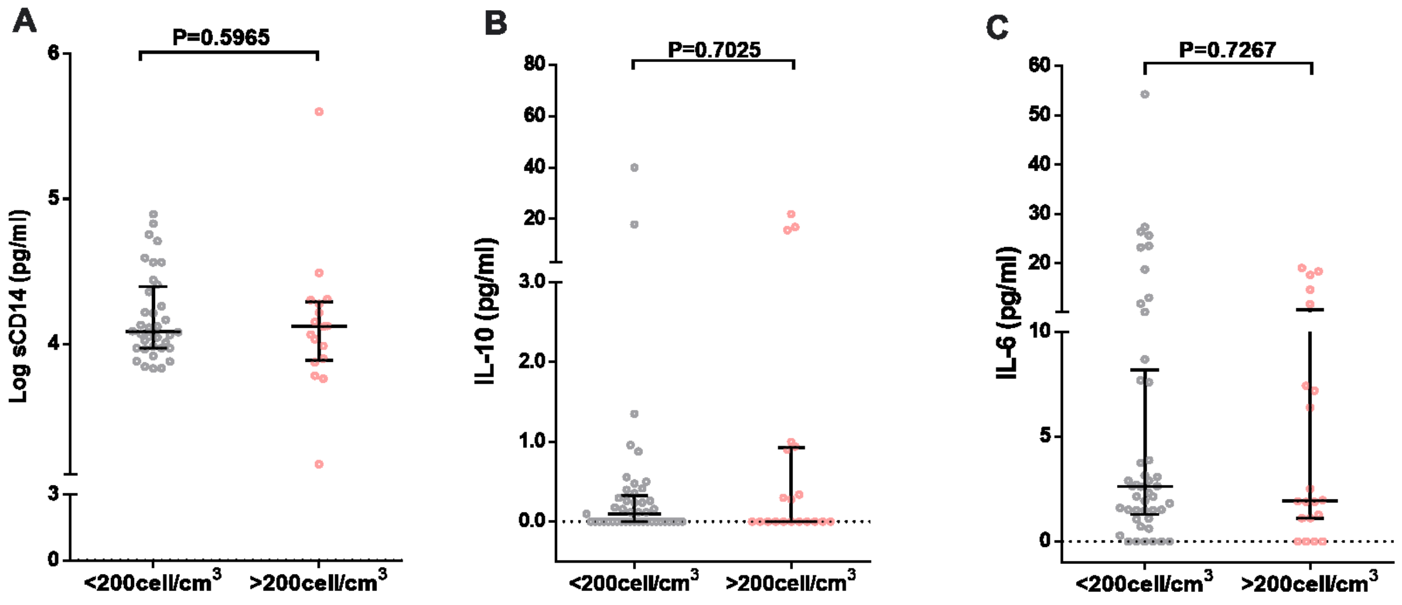


**Figure S1:** Effect of degree of baseline immunosuppression to plasma level of inflammatory and immune activation markers among virally suppressed HIV patients. Data depicts comparison of plasma levels of inflammatory or immune activation markers in patient with <200cells/cm$^3$ vs >200cells/cm$^3$ at baseline. A) Monocyte activation marker-sCD14, B) Interleukin-10 C) Interleukin-6. For comparison purposes IL-10 and IL-6 values below detection limit were regarded as zero (0). All P values was obtained by Mann–Whitney U test.
