# Supplementary material for: Advanced baseline immunosuppression is associated with elevated levels of plasma markers of fungal translocation and inflammation in long-term treated HIV-infected Tanzanians
Source: AIDS Res Ther. 2021 Aug 26;18:55. doi: 10.1186/s12981-021-00381-9 (PMC8394626; doi:10.1186/s12981-021-00381-9)
Supplement: Supplementary file 2 — Additional file 2: Table S1. Patients characteristics according to baseline CD4 counts [file 12981_2021_381_MOESM2_ESM.pdf]

Table S1: Patients characteristics according to baseline CD4 counts

Virally suppressed

	**Baseline CD4 count**		
	**<100 cells/cm^3 n=27**	**>100cells/cm^3 n=47**	**P-value**
**Demographic data**			
Median age in years (IQR)	48(43-54)	50(44-55)	0.2736[a]
Sex -female n (%)	24(88.8)	39(82.9)	0.4914[b]
**Clinical data**			
Median Years on treatment (IQR)	9.5(7-12)	9.5(5-12)	0.6634[a]
missing data	*n=1*	*n=7*	
Median current CD4 count cells/cm^3(IQR)	446(316-627)	547(408-635)	0.2248[a]
missing data	*n=1*		
Median proviral load copies/million PBMCs(IQR)	464.3(262.3-762.8)	513.7(317-912.2)	0.392[a]
Current ART Regime n (%)			0.3588[b]
NNRTI based first line regimen	22(81.5)	41(87.2)	
PI based second line regimen	5(18.5)	5(10.6)	
missing data		1(2.1)	
**Serology-median antibody titer(IQR)**			
Epstein Barr virus	10.6(10.10-10.80)	10.6 (7.4-10.8)	0.0962[a]
Cytomegalovirus	32(16-128)	16(16-64)	0.4748[a]

Untreated patients

	**Baseline CD4 count**		
	**<200 cells/cm^3 n=10**	**>200 cells/cm^3 n=30**	**P value**
Median age in years (IQR)	41(32-49)	36(31-44)	0.2168[a]
Sex -female n (%)	5(50)	22(73.3)	0.2464[c]
Median Viral load copies/ml (IQR)	102039 (37911-348357)	43204 (7043-155966)	0.1556[a]

[a] Chi square test, [b] Mann–Whitney U test, [c] Fisher's exact test

Abbreviations: IQR, interquartile range; ART, antiretroviral therapy; PBMCs, peripheral blood mononuclear cells.
